# Triboelectrification of Two-Dimensional Chemical Vapor Deposited WS_2_ at Nanoscale

**DOI:** 10.1038/s41598-019-49107-y

**Published:** 2019-08-29

**Authors:** He Wang, Chung-Che Huang, Tomas Polcar

**Affiliations:** 1 National Centre for Advanced Tribology, Faculty of Engineering and the Environment, University of Southampton, Southampton, SO17 1BJ UK; 2 Optoelectronics Research Centre, University of Southampton, Southampton, SO17 1BJ UK; 3 Department of Control Engineering, Faculty of Electrical Engineering, Czech Technical University in Prague, Technicka 2, 16627 Prague 6, Czech Republic

**Keywords:** Electronic devices, Electronic properties and materials, Two-dimensional materials

## Abstract

Triboelectric properties of chemical vapor deposited WS_2_ nanoflakes have been characterized in nano-range by atomic force microscopy (AFM) and Kelvin force microscopy (KFM). The triboelectric process is dependent on the thickness of WS_2_ nanoflakes, and it is sensitive to the adsorbates like water molecules, as well as transferred Pt from the tip on the sample. The density of tribo-charge can be modified by applying various biases to the conductive Pt-coated tip during the frictional process. Tunneling of the tribo-charge into the gap between WS_2_ and the underlying substrate results in a long lifetime, which is about 100 times longer than conventional triboelectric charges. Moreover, we observe a positive correlation between the layer number and resistance to charge dissipation. Our finding can become the driving force for a new category of two-dimensional (2D) WS_2_ triboelectrically controllable nanodevices.

## Introduction

WS_2_, a typical member of transition metal dichalcogenides (TMDs) group, possesses a similar structure to widely studied MoS_2_: W and S atoms are arranged to a sandwich structure by covalent bonds in the sequence of S-W-S for a single layer, whereas the bonding of neighboring sheets is relatively weak van der Waals interaction.

Investigation on WS_2_ just began in recent years owing to the lack of crystals in nature, but it is considered as one of the most promising TMDs, and potential applications in various fields such as catalytic^1^, lithium ion batteries^2^, solar cell active materials^3^ have been reported. It is expected to own the highest electron mobility among the semiconductive TMDs thanks to a reduced effective mass - mobility up to 234 cm^2^V^−1^s^−1^ in multilayered WS_2_ contacted with gold at room temperature has been reported^4^. Besides, the valence band splitting of single-layer WS_2_ is nearly three times larger than that of MoS_2_, which can facilitate the observation of valley Hall effect^5^. Also, optoelectronic devices with a relatively high quantum efficiency can be realized since the photoluminescence emission of WS_2_ is ~50 times higher than MoS_2_^6^. Furthermore, atomically-thin WS_2_ is known as n-type semiconductor (because of the numerous S vacancies) with a direct band-gap^7–9^ and field effect transistors based on vertical graphene/WS_2_ heterostructures exhibit a quite large on-state current and an unprecedentedly high on/off ratio of more than 10^6^ due to the combination of tunneling and thermionic transport^10^. In the meantime, WS_2_-based lubricant shows better thermal stability and oxidation resistance when compared to other TMDs^11–14^.

Triboelectrification, during which charges are transferred and accumulated on materials via frictional process, can occur during sliding, leading to a perturbation of the charge neutrality of WS_2_^15–18^. In this case, a charge may move through the material freely or be localized in specific atomic sites; thereby attractive or repulsive Columbic forces can arise, which can alter the frictional properties^19^. For low dimensional WS_2_ structures, the effect of tribo-charges could be either positive (accumulated charge could be transferred in the form of useful energy) or negative (an increase in friction, i.e. more energy dissipated during sliding). Therefore, it is imperative to conduct an investigation into the triboelectric properties of WS_2_, particularly in view of the poor understanding and extremely limited publications regarding this topic^20,21^.

In this study, high-quality WS_2_ nanofilms were grown by chemical vapor deposition (CVD) approach. Benefitting from the capability of accurately AFM-controlled triboelectric operation, the effect of bias voltages and the diffusion procedure were observed with AFM and KFM, which follows a similar path to previous publications^20,21^. However, the effects of layer number, moisture and applied rubbing force were also investigated; the correlation of material and charge transfer was reported for semiconductors like WS_2_ as well, since relevant results are only provided for insulating organic materials^22^. As the entire AFM apparatus can be integrated onto a microchip^23–25^, it is expected that triboelectrification-tuned systems and devices based on WS_2_ will come into reality in the near future.

## Materials and Methods

### Synthesis of WS_2_

All the WS_2_ nanoflakes were deposited on Si substrate with 300 nm SiO_2_ coating. As shown in Fig. S1, Supporting Information, the CVD reaction was operated in a 3 cm diameter quartz tube under atmospheric pressure. Prior to the reaction, SiO_2_/Si substrates were loaded on a boat containing 30 mg WO_3_ (Alfa Aescar 99.9995%) and 10 mg NaCl (Alfa Aescar 99.9995%, its addition can reduce the melting point of WO_3_ to facilitate the vaporization^26^), and then placed to the tube center. Afterward, another boat with 150 mg sulfur (Alfa Aescar 99.9995%) was placed at the upstream. After 10-minute purge with 300 standard cubic centimeters per minute (sccm) Ar gas (BOC, 99.999% pure with additional purifications), the flow rate was decreased to 30 sccm, and the furnace was subsequently programmed to 900 °C to heat substrates and vaporize WO_3_. In the meantime, sulfur was heated up to 200 °C by the heating tape so its vapor could be transported to the substrate. After 15 minutes of deposition, the furnace and heating band were switched off for natural cooling-down.

### Characterization of synthesized WS_2_

Optical microscope (Nikon LV200) and AFM (Scanning Probe Microscopy 5500, Agilent Technologies) were employed to measure the surface morphology of WS_2_ nanofilms. Raman spectroscopy (InVia Raman Spectrometer, excited with 532 nm laser) was utilized to characterize the vibrational modes, while scanning electrons microscopy (SEM, Zeiss EVO50XVP, with EDX system of Oxford Instruments INCA 250) and X-ray photoelectron spectroscopy (XPS, Thermo Scientific Theta Probe XPS System MC03, Al K *α* source) were operated to analyze the elemental composition.

### Generation and measurement of tribo-charges

Conductive probe (OMCL-AC240TM-R3 from Olympus, Pt-coated) was used for AFM and KFM. Firstly, contact-mode AFM was conducted at a 1 Hz scan rate to initiate tribo-charges on WS_2_ nanofilms, and various biases (−10~10 V) and normal forces (25~100 nN) were applied to the tip in some cases. KFM was subsequently performed with the tip biased by an AC voltage (amplitude: 0.8 V; frequency: 10 kHz) to obtain the surface potential maps.

To investigate the effect of surface treatment, WS_2_ nanoflakes were annealed at 100 °C for 10 minutes in some experiments. Once the heat treatment was completed, KFM measurements were conducted immediately to eliminate the environmental influence on the surface.

## Results and Discussion

**Figure 1:**
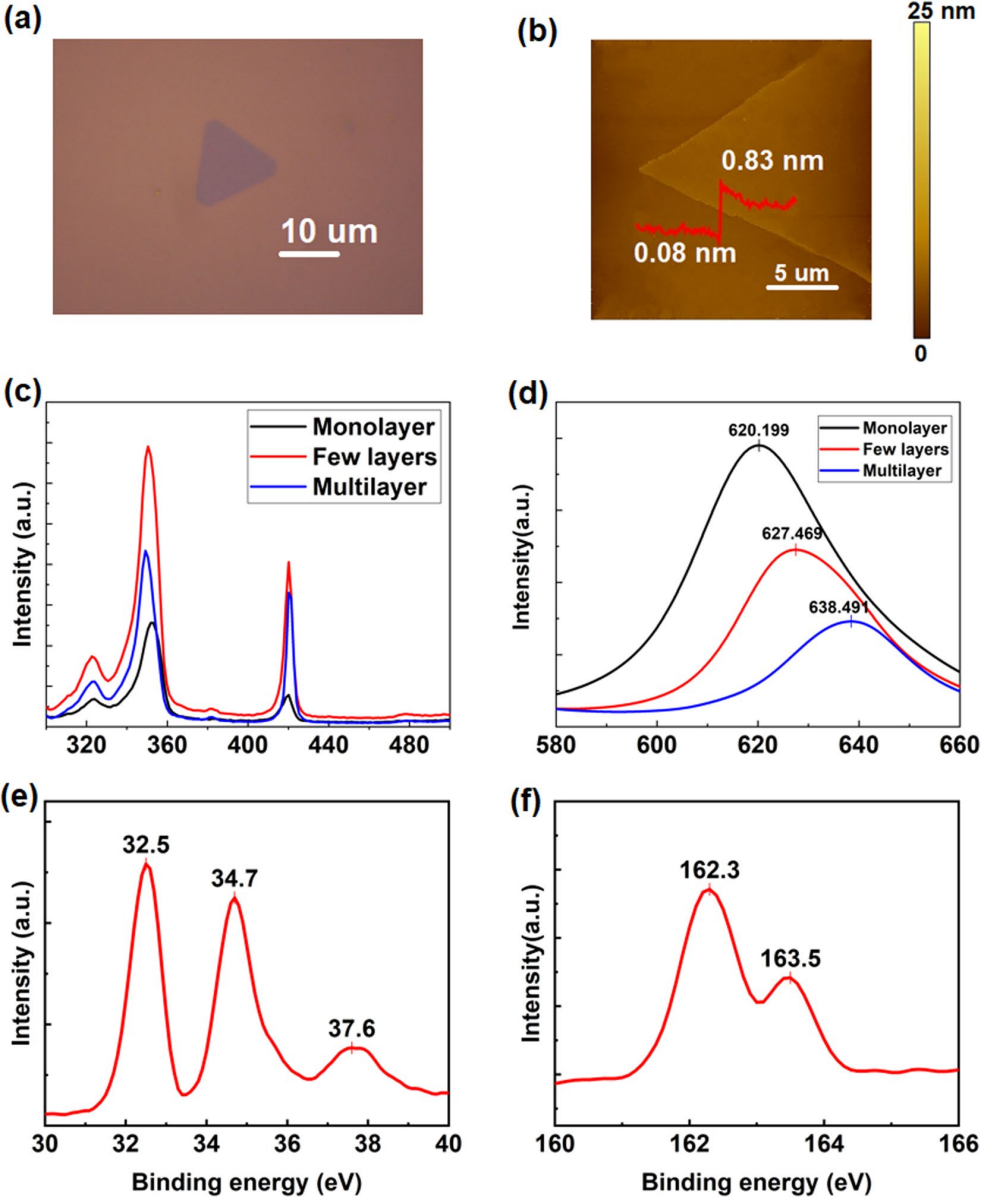
Characterization of WS_2_ nanoflakes synthesized on SiO_2_/Si substrate by CVD method. (**a**) Optical image and (**b**) Surface topographic map of a monolayer. (**c**) Raman spectrum and (**d**) PL spectrum with 532 nm laser excitation. XPS spectra of (**e**) W 4f and (**f**) S 2p orbitals of a monolayer.

The WS_2_ nanoflakes deposited on SiO_2_/Si substrate by CVD method were observed by optical microscopy, and their lateral size can reach up to ~100 µm as shown in Figs 1a and S2. Besides, the number of layers can be generally estimated via the variation in the color of nanoflakes on the substrate: while WS_2_ monolayer is almost transparent and shows a dark cyan color, a multilayer is brighter and a yellow color can be seen for much thicker flakes. To specify the exact layer number of different areas, surface topographies have been mapped by tapping-mode AFM, and the thickness is ~0.75 nm (Fig. 1b), which agrees with the reported thickness of monolayer WS_2_^27–29^. Apart from it, the surface topographic image of few layers and multilayers in Fig. S3 shows that their thicknesses are ~4.2 and ~7.1 nm corresponding to 5 layers and 9 layers, respectively. Besides, Raman spectrum was measured with 532 nm laser excitation for further characterization. As displayed in Fig. 1c, the 352.4 cm^−1^ 2LA peak and 419.6 cm^−1^ A_1g_ peak are obvious for monolayer, while a slight red shift of 2LA and a blue shift of A_1g_ mode were observed with the increase of layer number^30,31^. In addition, photoluminescence (PL) spectrum in Fig. 1d shows an obvious shift of emission peak from 620.2 to 638.5 nm, indicating the layer number of WS_2_ nanoflakes.

According to the XPS spectra of monolayer WS_2_ nanoflakes in Fig. 1e,f, the W 4f peaks at 34.7 and 32.5 eV are assigned to the 4f_5/2_ and 4f_7/2_ orbitals of WS_2_, respectively^32,33^; the W 5p core level at 37.6 eV suggests the existence of W^6+^, likely residual WO_3_ on the as-deposited sample^34^. 163.5 and 162.3 eV peaks belong to the S doublets: 2p_1/2_ and 2p_3/2_^32^. All the binding energies are indicative of WS_2_ crystal, and the atomic ratio of S and W is ~2:1. Apart from it, Fig. S4 displays the C 1 s orbital XPS spectrum: the 284.7 eV peak is the C-C bond, whose existence results from the use of carbon tapes to mount the sample on the sample-plate (The XPS spectrum was measured before the performance of AFM, so the C-C bond is not a result of AFM measurement); the C-S bond (in the range of 285–287.5 eV) cannot be detected, so the chemisorption of C is not significant^35^. Despite the fact that physisorption might happen, AFM working in contact mode could eliminate adsorbed molecules to the margins of rubbed regions^36^, so the surface topographies in Fig. S7, where there is no obvious materials accumulation on the margins of the rubbed area, further confirms the very limited contamination of the sample surface. Similar results were obtained for a few layers as well as multilayers, as displayed in Figs S5 and S6.

**Figure 2:**
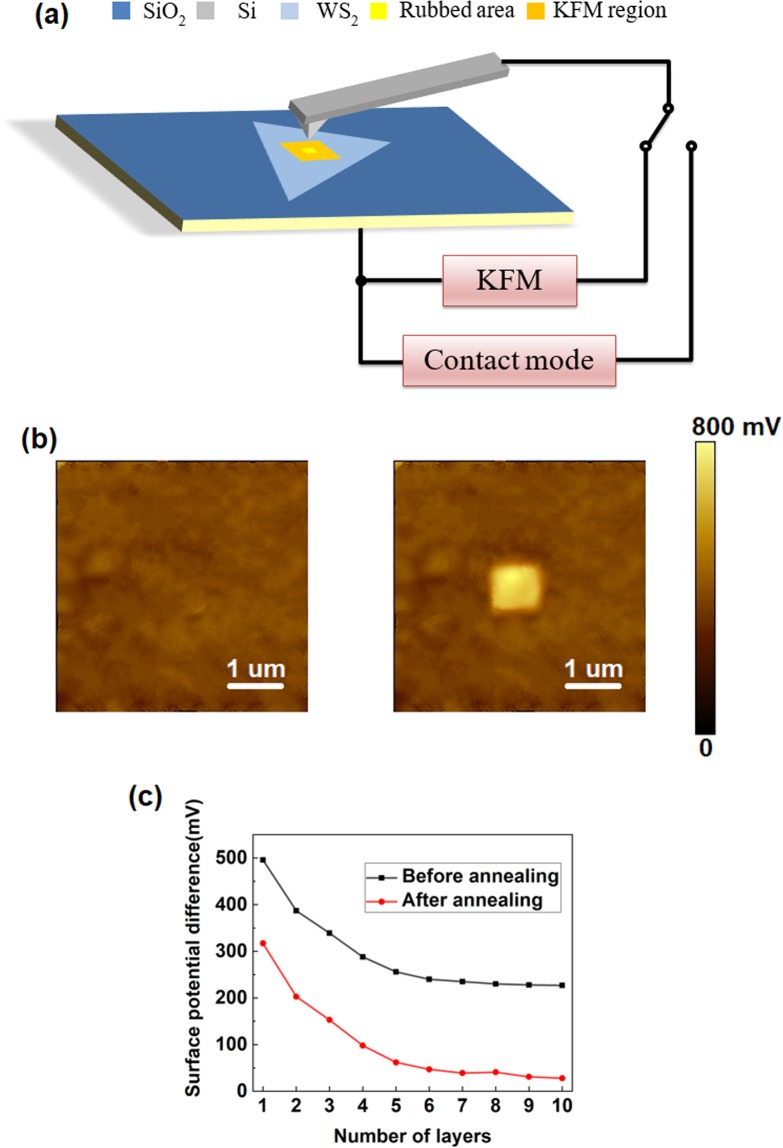
Characterization of triboelectric properties via AFM and KFM. (**a**) Schematic illustration of triboelectric experiments. (**b**) Surface potential images before and after the triboelectric process. (**c**) Surface potentials as a function of a number of WS_2_ layers before and after thermal treatment.

As demonstrated in Fig. 2a, AFM was performed in contact-mode to rub a 1 × 1 µm^2^ square region of the WS_2_ nanoflake by a conductive Pt-coated tip with 25-nN force applied normally, KFM mode was conducted to monitor the surface potential of a larger region (5 × 5 µm^2^), concentered with the rubbed region. From the surface potential maps (Fig. 2b), the single-layer WS_2_ is almost equipotential before triboelectrification, while the random fluctuations probably result from the adsorbed charges in the environment. After triboelectric process, the topographic variation remains unobvious (Fig. S7) but the surface potential of the rubbed region is ~0.5 V higher than that of the other region.

During the surface potential measurements, an AC voltage and a DC bias are applied between the tip and sample surface. The electrostatic forces acting on the conductive tip can be detected by a lock-in technique to monitor its oscillating amplitude within the system. The amplitude signal comprises the DC bias as well as the work function difference between the tip and sample, and the contact potential difference V_*CPD*_ is cancelled by the DC bias via a feedback circuit^37^, so their relationship can be described as Eq. 1:

1 $${{\rm{V}}}_{CPD}=\frac{1}{e}({\phi }_{tip}-{\phi }_{sample})$$

where *φ*_*tip*_ and *φ*_*sample*_ represent the work functions of the tip and sample, respectively; *e* is the electronic charge.

As a result, the contact potential difference between the rubbed and surrounding intact areas,ΔV_*CPD*_, can be computed as Eq. 2

2 $$\begin{array}{rcl}{\rm{\Delta }}{{\rm{V}}}_{CPD} & = & {{\rm{V}}}_{CPD}({\rm{rubbed}})-{{\rm{V}}}_{CPD}({\rm{unrubbed}})\\  & = & \frac{1}{e}({\phi }_{tip}-{\phi }_{rubbed})-\frac{1}{e}({\phi }_{tip}-{\phi }_{unrubbed})\\  & = & \frac{1}{e}({\phi }_{unrubbed}-{\phi }_{rubbed})\end{array}$$

where *φ*_*rubbed*_ and ${\phi }_{unrubbed}$ represent the work functions of the rubbed and unrubbed areas of the sample surface, respectively. As the Fermi levels of two materials are aligned when brought into contact, their work functions (the minimum energy needed to remove an electron from Fermi level to the vacuum immediately outside the solid surface) would be changed correspondingly. In this case, some electrons were transferred from the sample surface to the AFM tip via triboelectrification, leading to the enhancement of surface potential in the central region. With the increase of layer number, the surface potential becomes smaller, which can be explained by an interlayer screening effect, since the surface potential of a nanofilm is screened by the external electric field induced by the defects in the SiO_2_/Si substrate^38,39^.

It has been reported that the surface potential and the charge distribution of 2D materials, such as graphene and h-BN nanoflakes^40,41^, are extremely sensitive to the ambient atmosphere. For WS_2_, the water molecules adsorbed from the atmosphere may act as carrier trappers. It is suggested that defects on the SiO_2_/Si substrate mainly involves surface-adsorbed water molecules, so efficient heat treatment can reduce the presence of undesired adsorbents, thereby the effect of the external electric field from the substrate. To eliminate the effect of water, 10 minutes annealing at 100 °C was further performed on the rubbed WS_2_ nanoflakes, followed by immediate measurement of surface potential by KFM. As illustrated in Fig. 2c, while the surface potential difference still shows a similar descending tendency with the increase of layer number, the thermal treatment caused a decrease of ~0.2 mV. The reason behind is that the work function of unrubbed area (*φ*_*unrubbed*_) is lowered due to the removal of moisture via annealing, but the water molecules were already removed during the triboelectric process for rubbed central area, so the annealing has less influence on the work function of this area (*φ*_*rubbed*_). Based on Eq. 2, the difference of the contact potential between these two regions (ΔV_*CPD*_) is reduced.

**Figure 3:**
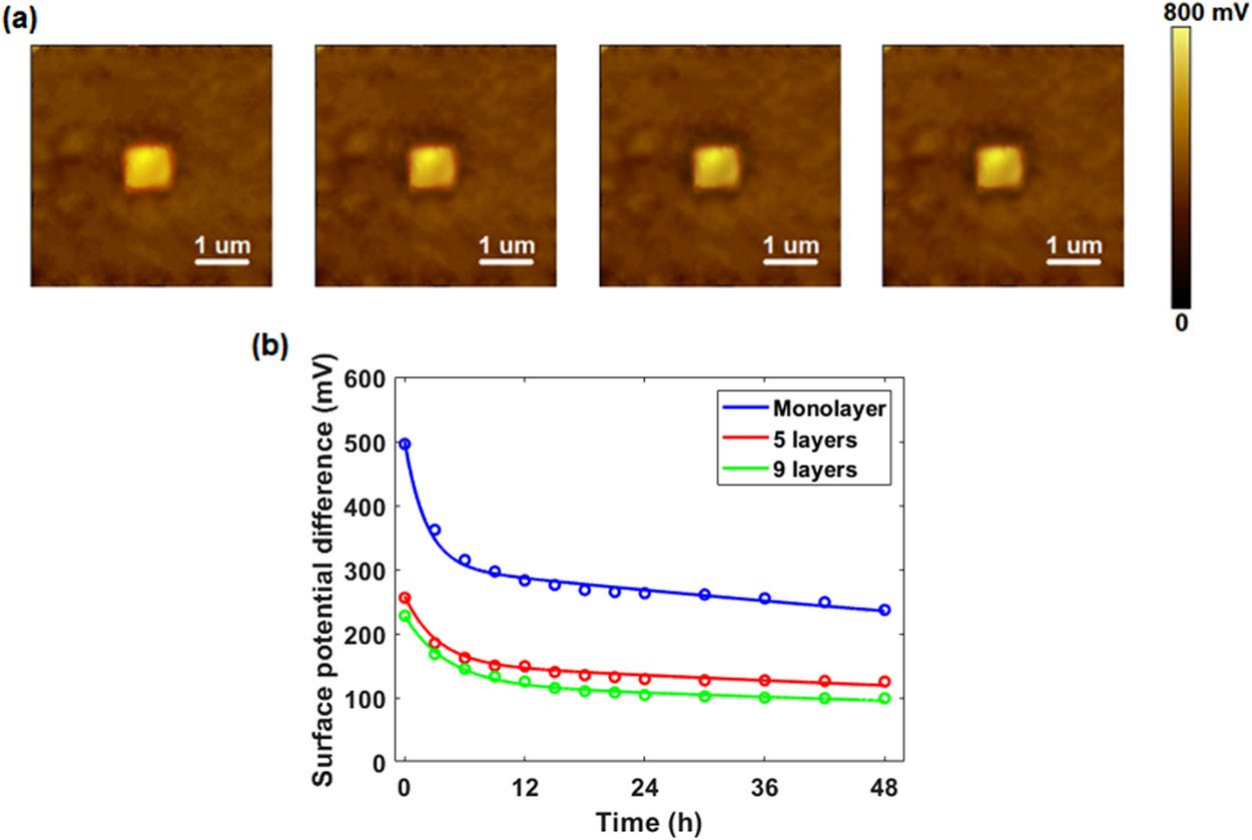
Diffusion of tribo-charges. (**a**) Surface potential maps after 12, 24, 36 and 48 hours. (**b**) Surface potential difference as a function of time and its fitted curve.

Figure 3a shows the surface potential maps after triboelectrification within 2 days. It can be noticed that the surface potential difference is still detectable even after 48 hours. Given that only insulating materials can store charges for that a long time, such observation cannot result from the charges on WS_2_. Here we introduce tunneling triboelectrification to define the tunneling of conventionally friction-induced charges (between WS_2_ and conductive tip) through WS_2_ nanosheets and their localization on the insulator (SiO_2_/Si substrate in this case) underneath^20,21^.

Here, it should be emphasized that the step height of monolayer WS_2_ measured by AFM (Fig. 1b), consists of a monolayer WS_2_ (a sandwich structure of S-W-S) and an air-gap at the interface. The lattice constant of a single-layer WS_2_ is ~0.3 nm^42^, so the air-gap between WS_2_ monolayer and the underlying SiO_2_/Si substrate can be calculated as 0.75–0.3 = 0.45 nm. The air-gap exists for WS_2_ nanoflakes with different layer numbers as well.

After triboelectric process, there should be no detectable current flow in WS_2_ once the equilibrium is reached, therefore, the discharge will only originate from leakage currents. As the diffusion of charges happens to both air and SiO_2_, two voltage magnitudes (*V*_1_,*V*_2_) and two time constants (*τ*_1_, *τ*_2_) are developed. Figure 3b shows surface potential difference (Δ*V*) with time for monolayer, 5 layers and 9 layers, and the fitted function is depicted below:

3 $${\rm{\Delta }}V={V}_{1}\times {e}^{\frac{-t}{{\tau }_{1}}}+{V}_{2}\times {e}^{\frac{-t}{{\tau }_{2}}}$$

All the parameters are listed in Table 1. It is obvious that apart from a term with a small magnitude (*V*_1_) and a short time constant (*τ*_1_), the other one has a larger initial amplitude (*V*_2_) and time constant (*τ*_2_). Since the air gap is considerably thinner than the SiO_2_ substrate, most charges are offered by WS_2_ nanoflakes and trapped at the interlayer, and a larger initial amplitude *V*_2_ is presented. The excellent insulation of air and the shield of WS_2_ nanofilms lead to a longer time constant *τ*_2_, which is ~200 times larger than the decaying time of tribo-charges on bare SiO_2_/Si substrate of the same thickness (~1 h)^43^. Considering the poor conductivity (higher work function) as well as better protection (more layers to shield the tunneling charges from the atmosphere) of multilayers, a larger time constant is observed as the thickness of nanoflakes increases.

**Table 1:** Parameters of fitted functions.

	*V*_1_/mV	*V*_2_/mV	*τ*_1_/h	*τ*_2_/h
Monolayer	194	305	2	184
5 layers	105	153	3	191
9 layers	106	121	4	199

**Figure 4:**
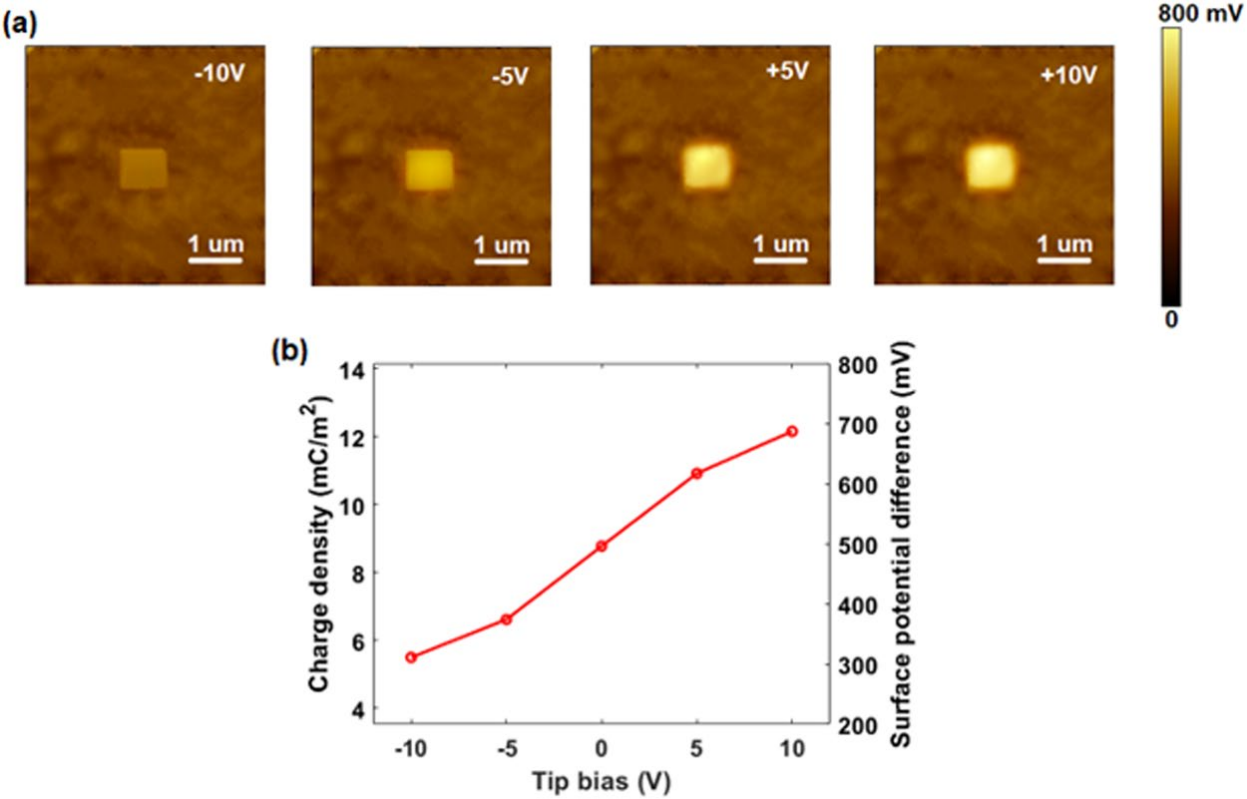
Controllable density of tribo-charges via bias voltage. (**a**) Surface potential maps after triboelectric process with different biases: −10, −5, +5 and +10 V. (**b**) Charge density and surface potential difference with tip bias.

Taking advantage of the versatile AFM systems, we can tune the density of tribo-charges on WS_2_ nanoflakes via various biases onto the conductive tip during the sliding process. As evident from Fig. 4a, different surface potentials can be measured for the rubbed region, indicating that the amount of tribo-charges localized on the sample surface is controllable.

Using the capacitor model^20,21^, the density of tribo-charges (σ) can be calculated as Eq. 4:

4 $$\sigma =\frac{{\rm{\Delta }}V\,{\varepsilon }_{0}{\varepsilon }_{Air}}{{t}_{Air}}$$

where Δ*V* is the potential difference, *ε*_0_ is the permittivity of vacuum,*t*_*Air*_ and *ε*_*Air*_ are the thickness and relative permittivity of the air gap, respectively.

Figure 4b demonstrates that the charge density, which is proportional to surface potential, increases linearly with the bias voltage, confirming the controllable density of tribo-charges via varied biases.

**Figure 5:**
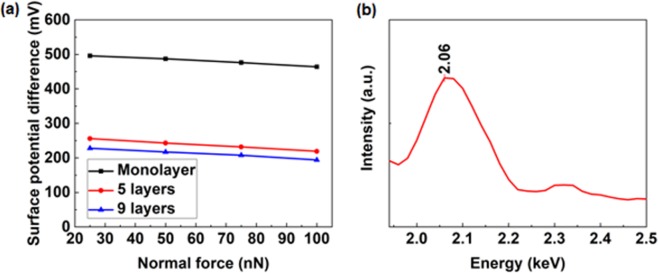
Material transfer during triboelectric process. (**a**) Surface potentials with different applied normal forces. Monolayer (1 layer), few layer (5 layers) and multilayer (9 layers). (**b**) EDX map of rubbed area for monolayer WS_2_ applied with 25 nN normal force to show the Pt peak.

AFM system is capable of scanning with varied normal forces and the as-grown WS_2_ nanoflakes were rubbed with 25, 50, 75 and 100 nN to investigate the effect of load. As evident from the surface potential difference shown in Fig. 5a, there is a slight reduction in the surface potential differences for monolayer, few layer and multilayer with the increase of applied normal force. However, the work function should be identical for the measured nanoflakes with the same thickness, so it is suggested that some materials may be transferred during the rubbing process. Then, elemental composition of the rubbed areas was analyzed by EDX, and an energy peak at 2.06 keV, belonging to Pt, is detected in Fig. 5b.

The chemical composition of rubbed WS_2_ monolayer with various applied normal forces is listed in Table 2. The atomic ratio of W and S is ~1:2, which is consistent with the XPS results in Fig. 1, and Pt content increased with applied force. Note that low values of chamical composition related to Pt, W and S are due to EDX interaction volume (max. depth approx. 1 μm for acceleration voltage 20 kV).

**Table 2:** Atomic ratios of different elements on monolayer with various applied normal forces.

Normal force/nN	Pt	Si	C	O	W	S
25	0.24	31.55	1.18	66.73	0.10	0.20
50	0.33	31.24	1.01	67.14	0.09	0.19
75	0.39	30.97	0.98	67.37	0.09	0.20
100	0.44	30.80	0.91	67.58	0.08	0.19

Obviously, there is a slight rise in Pt ratio with the increase of the applied forces. Considering the negative correlation between the surface potential difference and applied force, we suppose that it is Pt that has been transferred from the tip to the WS_2_ nanoflakes, since the transfer of Pt (with a larger work function) can enhance the work function of WS_2_, thereby lowering the surface potential difference as depicted by Eq. 2. Based on this property, WS_2_ can be utilized as an interlayer for charge storage in triboelectric nanogenerators to promote the output performance, as reported for MoS_2_^44^. Besides, WS_2_ can be applied to triboelectrically controllable devices and systems, such as transistors, photonics, detectors and antennas to name a few.

## Conclusions

In summary, nanoscale triboelectrification of chemical vapor deposited WS_2_ nanoflakes has been investigated with the help of AFM as well as KFM. Charge transfer process and the effect of temperature were observed systematically. We show that the surface potential becomes smaller with the increase of thickness, which originates from the interlayer screening effect of external electric field induced by the defects in the underlying substrate, and heat treatment can mitigate this effect efficiently by removing undesired adsorbents. The diffusion of triboelectric charges on WS_2_ nanoflakes was then monitored, and it is found that these tribo-charges can be stored for an extremely long period of time, and the dwell time is positively correlated to the layer number. Furthermore, different amount of tribo-charges can be initiated by applying various biases onto the conducting tip during the triboelectric process. At last, the effect of applied normal force during the rubbing process was studied. Some Pt has been transferred from the tip to the WS_2_ nanoflakes to tune its work function. These distinctive properties provide a guidance for the design of WS_2_-based triboelectrification-controlled nanodevices, such as nanogenerators, photonics, transistors or dividers.

## Supplementary information

Triboelectrification of Two-dimensional Chemical Vapor Deposited WS<sub>2</sub> at Nanoscale

## References

[1] Lukowski Mark A., Daniel Andrew S., English Caroline R., Meng Fei, Forticaux Audrey, Hamers Robert J., Jin Song (2014). Highly active hydrogen evolution catalysis from metallic WS2 nanosheets. Energy Environ. Sci..

[2] Armstrong Mark J., O’Dwyer Colm, Macklin William J., Holmes Justin. D. (2013). Evaluating the performance of nanostructured materials as lithium-ion battery electrodes. Nano Research.

[3] Shanmugam Mariyappan, Bansal Tanesh, Durcan Chris A., Yu Bin (2012). Schottky-barrier solar cell based on layered semiconductor tungsten disulfide nanofilm. Applied Physics Letters.

[4] Liu Xue, Hu Jin, Yue Chunlei, Della Fera Nicholas, Ling Yun, Mao Zhiqiang, Wei Jiang (2014). High Performance Field-Effect Transistor Based on Multilayer Tungsten Disulfide. ACS Nano.

[5] Ovchinnikov Dmitry, Allain Adrien, Huang Ying-Sheng, Dumcenco Dumitru, Kis Andras (2014). Electrical Transport Properties of Single-Layer WS2. ACS Nano.

[6] Peimyoo N (2013). Nonblinking, intense two-dimensional light emitter: Monolayer WS2 Triangles. ACS Nano.

[7] Iqbal, M. W. *et al*. High-mobility and air-stable single-layer WS2 field-effect transistors sandwiched between chemical vapor deposition-grown hexagonal BN films. *Sci*. *Rep*., 10.1038/srep10699 (2015). 10.1038/srep10699 PMC4450543 26030008

[8] Gutiérrez Humberto R., Perea-López Nestor, Elías Ana Laura, Berkdemir Ayse, Wang Bei, Lv Ruitao, López-Urías Florentino, Crespi Vincent H., Terrones Humberto, Terrones Mauricio (2012). Extraordinary Room-Temperature Photoluminescence in Triangular WS2 Monolayers. Nano Letters.

[9] Shang Jingzhi, Shen Xiaonan, Cong Chunxiao, Peimyoo Namphung, Cao Bingchen, Eginligil Mustafa, Yu Ting (2015). Observation of Excitonic Fine Structure in a 2D Transition-Metal Dichalcogenide Semiconductor. ACS Nano.

[10] Georgiou, T. *et al*. Vertical Field Effect Transistor based on Graphene-WS2 Heterostructures for flexible and transparent electronics. 10.1038/nnano.2012.224 23263726

[11] Feng Dongdong, Peng Jinfeng, Liu Sisi, Zheng Xuejun, Yan Xinyang, He Wenyuan (2018). Influences of thickness, scanning velocity and relative humidity on the frictional properties of WS2 nanosheets. Materials Research Express.

[12] Boesiger, E. A. 44th Aerospace Mechanisms Symposium. *Aerosp*. *Mech*. *Symp*. **44** (2018).

[13] Watanabe S, Noshiro J, Miyake S (2004). Tribological characteristics of WS2/MoS2solid lubricating multilayer films. Surf. Coatings Technol..

[14] Sliney HE (1982). Solid lubricant materials for high temperatures-a review. Tribol. Int..

[15] Burgo, T. A. L., Silva, C. A., Balestrin, L. B. S. & Galembeck, F. Friction coefficient dependence on electrostatic tribocharging. *Sci*. *Rep*., 10.1038/srep02384 (2013). 10.1038/srep02384 PMC3740278 23934227

[16] LOEB L. B. (1945). THE BASIC MECHANISMS OF STATIC ELECTRIFICATION. Science.

[17] Diaz A.F., Felix-Navarro R.M. (2004). A semi-quantitative tribo-electric series for polymeric materials: the influence of chemical structure and properties. Journal of Electrostatics.

[18] Fan Feng Ru, Tang Wei, Wang Zhong Lin (2016). Flexible Nanogenerators for Energy Harvesting and Self-Powered Electronics. Advanced Materials.

[19] Cammarata A, Polcar T (2017). Vibrational contributions to intrinsic friction in charged transition metal dichalcogenides. Nanoscale.

[20] Kim S (2017). Rewritable ghost floating gates by tunnelling triboelectrification for two-dimensional electronics. Nat. Commun..

[21] Wang H, Huang C-C, Polcar T (2019). Controllable Tunneling Triboelectrification of Two-Dimensional Chemical Vapor Deposited MoS2. Sci. Rep..

[22] Pandey RK, Kakehashi H, Nakanishi H, Soh S (2018). Correlating Material Transfer and Charge Transfer in Contact Electrification. J. Phys. Chem. C.

[23] Barrettino D (2005). CMOS monolithic mechatronic microsystem for surface imaging and force response studies. in. IEEE Journal of Solid-State Circuits.

[24] Chu LL, Takahata K, Selvaganapathy PR, Gianchandani YB, Shohet JL (2005). A micromachined Kelvin probe with integrated actuator for microfluidic and solid-state applications. J. Microelectromechanical Syst..

[25] Vettiger P (2002). The ‘Millipede’ - Nanotechnology Entering Data Storage. IEEE Trans. Nanotechnol..

[26] Zhou J (2018). A library of atomically thin metal chalcogenides. Nature.

[27] Liu P (2017). Large-Area WS2 Film with Big Single Domains Grown by Chemical Vapor Deposition. Nanoscale Res. Lett..

[28] Hussain, A. M., Sevilla, G. A. T., Rader, K. R. & Hussain, M. M. Chemical vapor deposition based tungsten disulfide (WS2) thin film transistor. 2013 *Saudi Int*. *Electron*. *Commun*. *Photonics Conf*. *SIECPC* 2013, 10.1109/SIECPC.2013.6550981 (2013).

[29] Fu Q (2015). Controllable synthesis of high quality monolayer WS2 on a SiO2/Si substrate by chemical vapor deposition. RSC Adv..

[30] Lv Y (2018). Preparation and Photoluminescence of Tungsten Disulfide Monolayer. Coatings.

[31] Qiao, S., Yang, H., Bai, Z., Peng, G. & Zhang, X. Identifying the number of WS2 layers via Raman and photoluminescence spectrum. **141**, 1408–1413 (2017).

[32] Martinez H (2004). Influence of the cation nature of high sulfur content oxysulfide thin films MOySz (M = W, Ti) studied by XPS. Appl. Surf. Sci..

[33] Cattelan Mattia, Markman Brian, Lucchini Giacomo, Das Pranab Kumar, Vobornik Ivana, Robinson Joshua Alexander, Agnoli Stefano, Granozzi Gaetano (2015). New Strategy for the Growth of Complex Heterostructures Based on Different 2D Materials. Chemistry of Materials.

[34] Reale, F. Chemical Vapour Deposition of Atomically Thin Tungsten Disulphide. (2017).

[35] Bratt, A. & Barron, A. R. XPS of Carbon Nanomaterials. *Measurement* 1–16 (2011).

[36] Zekonyte J, Polcar T (2015). Friction Force Microscopy Analysis of Self-Adaptive W-S-C Coatings: Nanoscale Friction and Wear. ACS Appl. Mater. Interfaces.

[37] Nonnenmacher M, O’Boyle MP, Wickramasinghe HK (1991). Kelvin probe force microscopy. Appl. Phys. Lett..

[38] Lee N. J., Yoo J. W., Choi Y. J., Kang C. J., Jeon D. Y., Kim D. C., Seo S., Chung H. J. (2009). The interlayer screening effect of graphene sheets investigated by Kelvin probe force microscopy. Applied Physics Letters.

[39] Li Yang, Xu Cheng-Yan, Zhen Liang (2013). Surface potential and interlayer screening effects of few-layer MoS2 nanoflakes. Applied Physics Letters.

[40] Ziegler, D. *et al*. Variations in the work function of doped single- and few-layer graphene assessed by Kelvin probe force microscopy and density functional theory. *Phys*. *Rev*. *B - Condens*. *Matter Mater*. *Phys*., 10.1103/PhysRevB.83.235434 (2011).

[41] Oliveira C K, Matos M J S, Mazzoni M S C, Chacham H, Neves B R A (2012). Anomalous response of supported few-layer hexagonal boron nitride to DC electric fields: a confined water effect?. Nanotechnology.

[42] Reale F (2017). High-Mobility and High-Optical Quality Atomically Thin WS2. Sci. Rep..

[43] Zhou YS (2013). *In situ* quantitative study of nanoscale triboelectrification and patterning. Nano Lett..

[44] Wu Chaoxing, Kim Tae Whan, Park Jae Hyeon, An Haoqun, Shao Jiajia, Chen Xiangyu, Wang Zhong Lin (2017). Enhanced Triboelectric Nanogenerators Based on MoS2 Monolayer Nanocomposites Acting as Electron-Acceptor Layers. ACS Nano.

