# Supplementary material for: Triboelectrification of Two-Dimensional Chemical Vapor Deposited WS2 at Nanoscale
Source: Sci Rep. 2019 Aug 29;9:12570. doi: 10.1038/s41598-019-49107-y (PMC6715710; doi:10.1038/s41598-019-49107-y)

Supporting Information

Triboelectrification of Two-dimensional Chemical Vapor Deposited WS_2 at Nanoscale

He Wang, Chung-Che Huang, Tomas Polcar

Fig. S1 Schematic illustration for the growth of WS_2 films on the SiO_2/Si substrates by CVD.

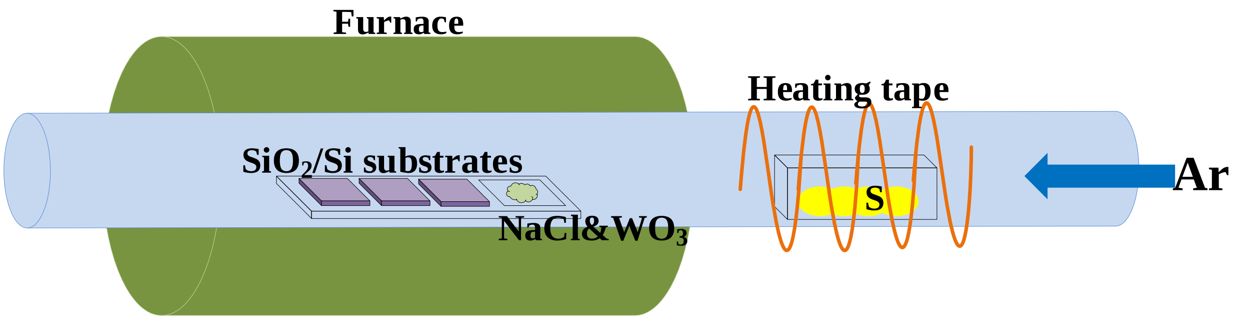

Fig. S2 Optical image of few layers (5 layers) and multilayers (9 layers).

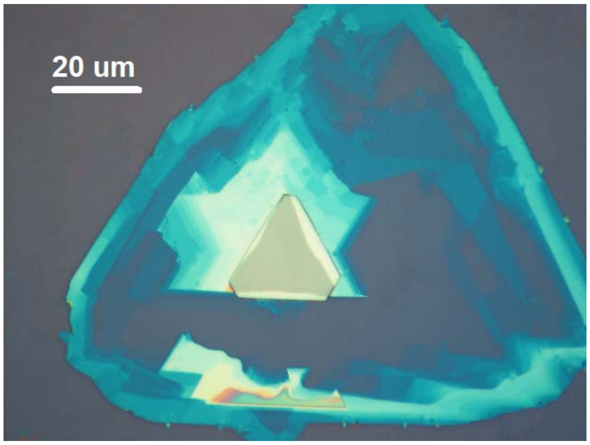

Fig. S3 Surface topography of few layers and multilayers.

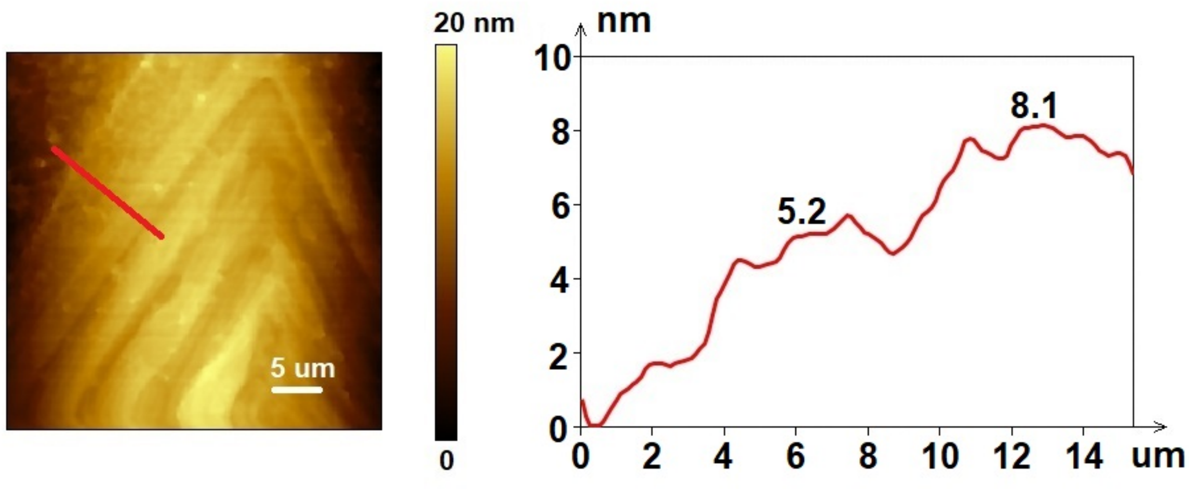

Fig. S4 XPS spectrum of C 1s orbital (monolayer).

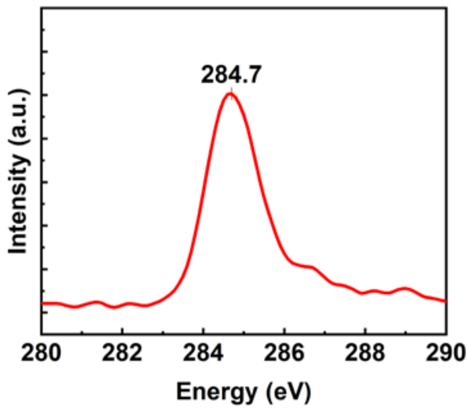

Fig. S5 XPS spectra of (a) W 4f, (b) S 2p and (c) C 1s orbitals of few layers.

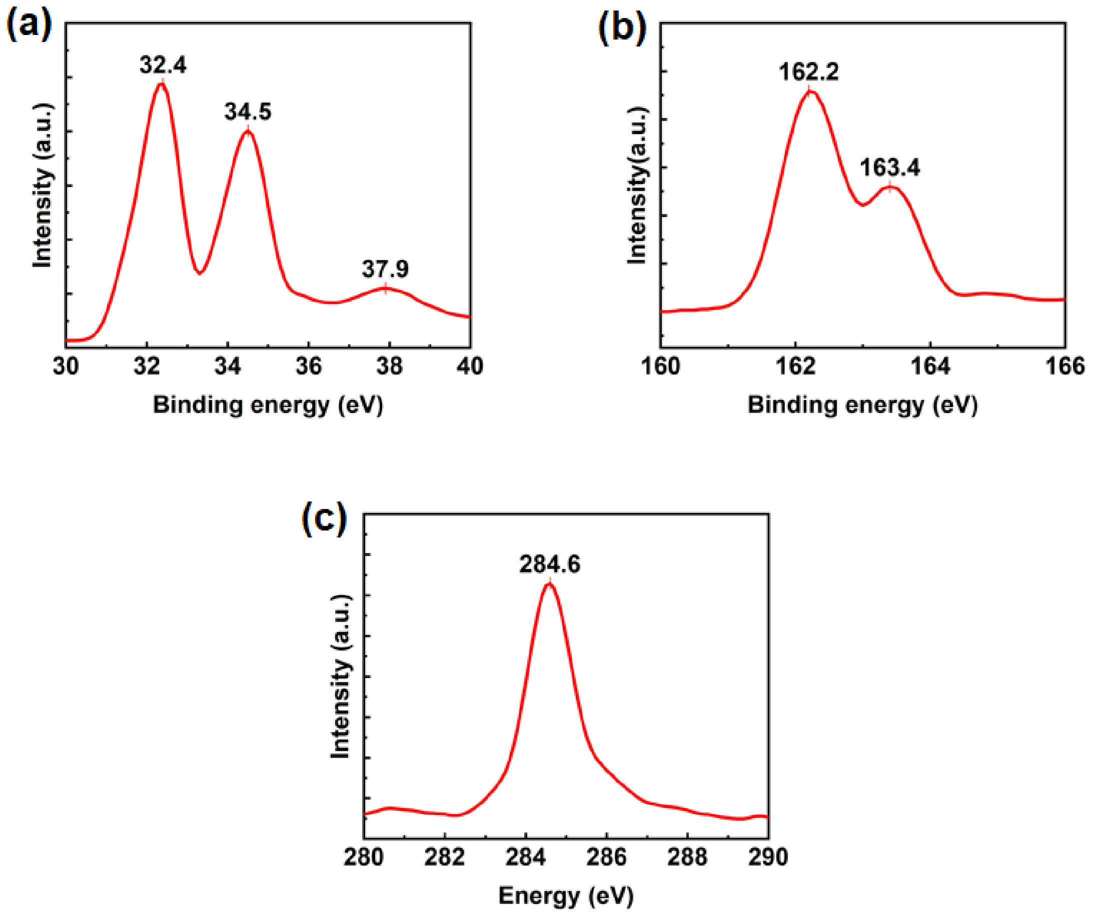

Fig. S6 XPS spectra of (a) W 4f, (b) S 2p and (c) C 1s orbitals of multilayers.

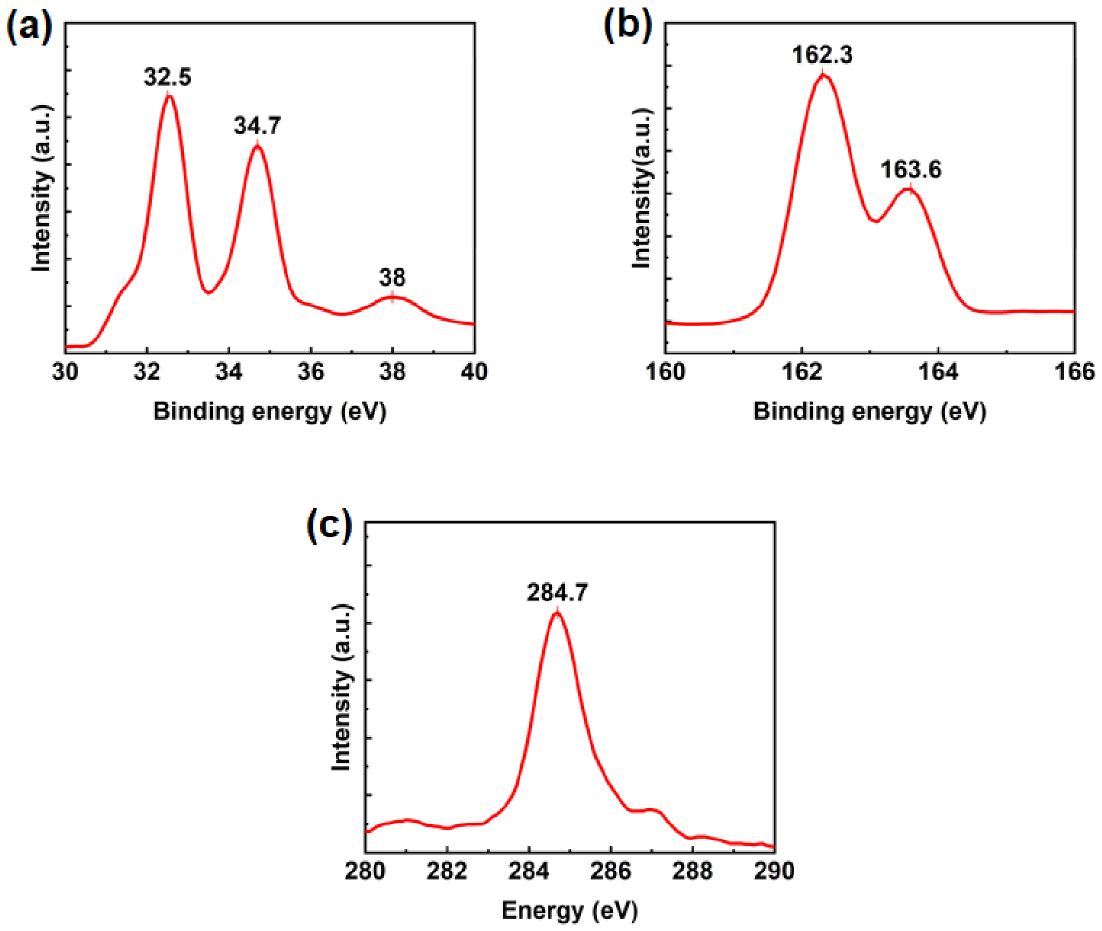

Fig. S7 Surface topographies before and after triboelectrification, with rubbed region squared.

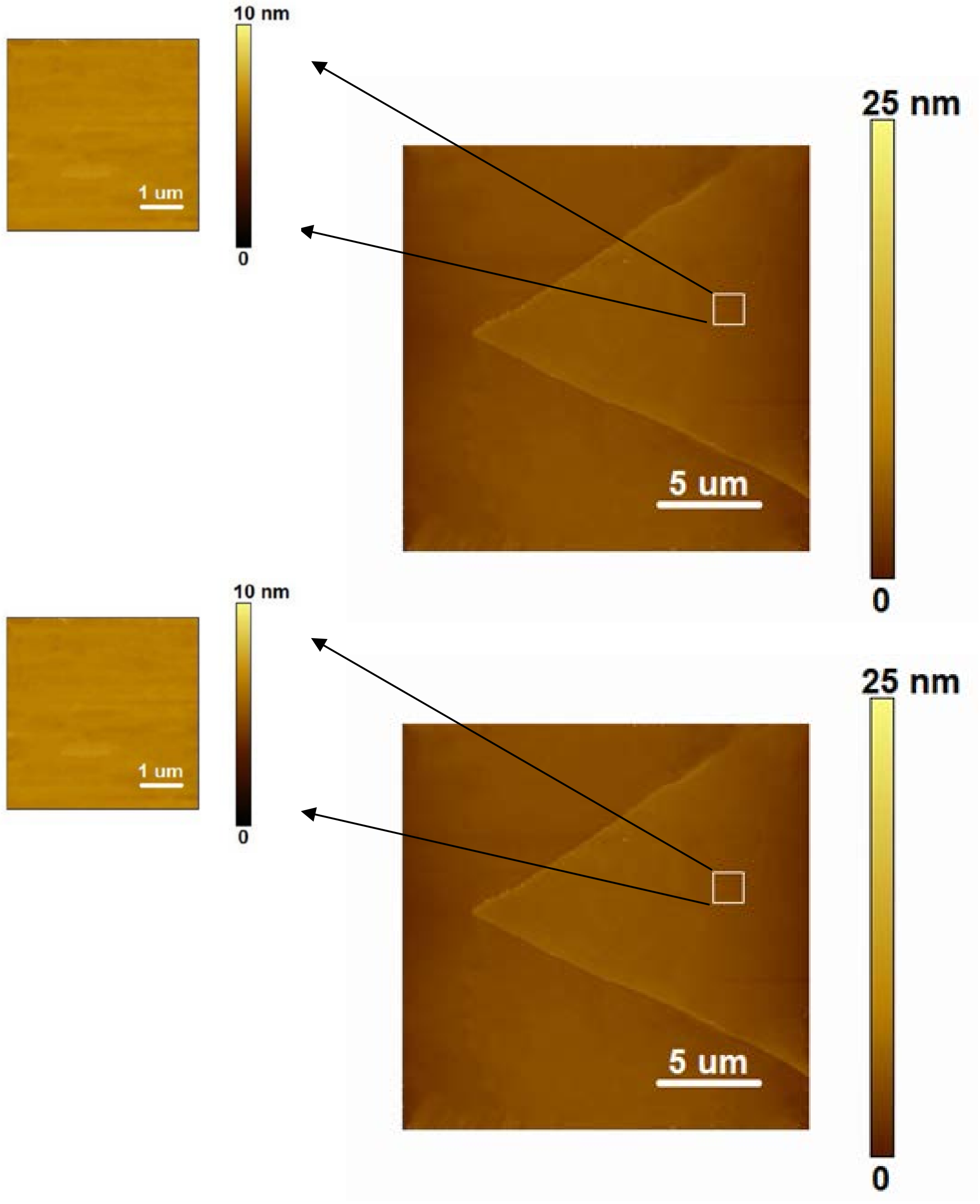

Supplement: Supplementary file 1 — Triboelectrification of Two-dimensional Chemical Vapor Deposited WS2 at Nanoscale [file 41598_2019_49107_MOESM1_ESM.pdf]
